# Do self‐reports of procrastination predict actual behavior?

**DOI:** 10.1002/mpr.1843

**Published:** 2020-06-12

**Authors:** Sascha Zuber, Stéphanie Cauvin, Maximilian Haas, Anne‐Sophie Daviet, Chloé Da Silva Coelho, Matthias Kliegel

**Affiliations:** ^1^ Center for the Interdisciplinary Study of Gerontology and Vulnerability University of Geneva Geneva Switzerland; ^2^ Swiss National Centre of Competences in Research, LIVES–Overcoming Vulnerability: Life Course Perspectives Lausanne and Geneva Switzerland; ^3^ Department of Psychology University of Geneva Geneva Switzerland

**Keywords:** procrastination, pure procrastination scale, self‐assessment, self‐reports, validation

## Abstract

**Objectives:**

Procrastination is typically assessed via self‐report questionnaires. So far, only very few studies have examined actual procrastination behavior, providing inconclusive results regarding the real‐life validity of self‐reports in this domain. The present study aimed to examine for the first time whether participants' self‐reported procrastination can predict their actual behavior on a real‐life task.

**Methods:**

For that purpose, we assessed self‐reported levels of procrastination [via the Pure Procrastination Scale, PPS] and actual procrastination behavior on a naturalistic task [i.e., having to send in an attendance sheet before a deadline] in 93 participants.

**Results:**

Results show that self‐reports significantly predicted procrastination behavior. Analyses of underlying dimensions suggest that real‐life procrastination can be the result of “voluntarily delaying planned actions,” but can also have more passive causes such as “running out of time.”

**Conclusions:**

Comparing our results with the available literature suggests that PPS self‐reports reflect a particularly valid tool to assess real‐life procrastination behavior. Findings are discussed in the context of strategies and mechanisms that potential interventions may target in order to reduce procrastination.

## 1 INTRODUCTION

Procrastination can be defined as intentionally putting something off or voluntarily delaying an intended course of actions although one expects negative consequences due to this delay (Ferrari, 2001; Steel, Brothen, & Wambach, 2001). Procrastination forms a prevalent topic in modern society, and consequently has been studied extensively over the last two decades: 20% of the adult population indicate being chronically affected by procrastination (Harriott & Ferrari, 1996), whereas almost 50% of students report that they frequently procrastinate (Steel, 2007). Studies show that procrastination has a negative impact on our daily life, as it is associated to lower life satisfaction and personal well‐being (Balkis & Duru, 2016; Tice & Baumeister, 1997), to higher financial problems (Steel, 2007), to less career success (Mehrabian, 2000), and to increased health problems (Sirois, 2007; Sirois, Melia‐Gordon, & Pychyl, 2003).

So far, researchers have predominantly used self‐report questionnaires to study procrastination and its correlates (Kim & Seo, 2015). Consequently, there is a variety of questionnaires allowing to interrogate participants on their procrastination habits, each studying different aspects of procrastination (Svartdal & Steel, 2017). With the objective of synthesizing such questionnaires and better understanding the constructs that underlie procrastination, Steel (2010) conducted a large‐scale study on over 4,000 participants and subsequently presented the Pure Procrastination Scale [PPS], a concise 12‐item questionnaire evaluating participants' general level of procrastination. The PPS allows to distinguish between two dimensions of procrastination, namely, “voluntary delay” and “observed delay” (see Rebetez, Rochat, Gay, & Van der Linden, 2014). Voluntary delay describes actively choosing to postpone certain actions or decisions, voluntarily putting off certain tasks, or preferring doing certain things later while ignoring potential negative consequences. In contrast, observed delay [also labeled “lateness” or “timeliness”] (see Svartdal & Steel, 2017) comprises a more passive notion of generally not being able to meet deadlines or frequently running out of time (Rebetez et al., 2014).

Different studies show that the PPS represents a valid instrument, which may measure self‐reported procrastination more accurately and more consistently than other questionnaires (Rebetez et al., 2014; Steel, 2010; Svartdal, 2017; Svartdal et al., 2016; Svartdal & Steel, 2017). However, to this day, there are no studies that have investigated whether PPS self‐reports correlate with participants' actual behavior. In fact, there is a general paucity of research using behavioral indicators of procrastination, as previous studies have mostly relied on participants' self‐reports. This is an important limitation of the available literature, as self‐reports do not necessarily only reflect actual behavior but can also be influenced by factors such as self‐esteem and self‐perception [e.g., labeling oneself as “procrastinator” because of low self‐esteem rather than due to actual behavior] or emotional valence of past events [e.g., remembering one occasion where procrastination had a particularly negative outcome]. Rotenstein, Davis, and Tatum (2009), for example, have pointed out that “self‐reported measures are often weak measures of actual procrastination” [p. 224]. Similarly, Krause and Freund (2014) state that “how well scale‐based self‐report measures of procrastination reflect the actual behavior remains subject of an on‐going debate, and is currently understudied” [p. 75].

So far, there are only seven studies that address the relation between self‐reports and behavior explicitly, and only three address onset delay specifically (Moon & Illingworth, 2005; Senecal, Lavoie, & Koestner, 1997; Steel et al., 2001; see Svartdal, Granmo, & Færevaag, 2018, for a recent overview). These studies either did not find a significant association between self‐reports and behavior or they observed small to moderate correlations between the two domains [typically between .10 and .30] (see Krause & Freund, 2014). Importantly, even in this line of research, the majority of the studies did not use concrete and objective measures of procrastination behavior that appropriately reflect typical real‐life procrastination [such as “how many days someone waited before performing a particular action”]. Instead, they mostly referred to other means of assessing procrastination, such as self‐reports on participants' behavior [i.e., participants had to report how many planned actions they have postponed], which are still prone to the biases mentioned above, or they used more atypical measures which do not necessarily reflect behaviors that one would typically see as procrastinating [e.g., observing whether participants were “walking” vs. “standing” while taking an escalator and using “standing” as an indicator of procrastination].

Taken together, the currently available literature on the relation of self‐reported and behavioral procrastination remains scarce, inconsistent, and inconclusive. With the present study, we aimed to examine the relation between self‐reports and procrastination behavior in more detail by using an objective external criterion behavior [namely, how many days participants delayed an intended action before performing this particular action]. Thus, the first goal of the present study was to investigate whether self‐reports assessed via the PPS represent an appropriate proxy of people's actual procrastination behavior. If such were the case, for clinicians and researchers, this would have the advantage that procrastination could be assessed reliably by using a short and easy‐to‐administer questionnaire rather than having to administer more time‐ and effort‐consuming behavioral procrastination tasks.

In addition, as the PPS allows disentangling the two dimensions of procrastination, the second goal of the present study was to examine whether participants' behavior is more strongly predicted by their tendency to actively delay actions [voluntary delay], whether it is more strongly linked to the passive notion of not completing tasks [observed delay], or whether both dimensions similarly relate to procrastination behavior. This is of theoretical as well as of clinical relevance. On the one hand, it will show whether procrastination behavior relates to one or both types of self‐perceived procrastination and thus will provide novel insights on the mechanisms that underlie procrastination. On the other hand, it may also provide helpful insight and suggest new directions for clinicians and researchers by illustrating which of these dimensions should be targeted when assessing procrastination or when developing future interventions that aim to reduce procrastination. For example, differential associations to procrastination subfacets could indicate whether interventions should rather focus on the voluntary postponement of actions [e.g., training to immediately perform actions as soon as an opportunity to do so occurs] or whether such programs should rather target the aspect of observed delay [e.g., training procrastinators' planning and organization skills or targeting motivation and emotion regulation].

## 2 METHOD

### 2.1 Sample size and power

For a medium effect‐size, a two‐sided alpha of .05 and a power of 0.95, the required minimum sample size was of 89 participants.

### 2.2 Participants

The current sample includes 93 students [*M* = 23.16, *SD* = 4.85; age range = 19–47 years; 12 men; one participant was excluded due to missing data], who participated in the study in exchange for course credits of a mandatory course in the second‐year Bachelor's program. All participants who were included in subsequent analyses still required those course credits. All participants were students at the University of Geneva and either spoke French as first language or had an equivalent level of fluency. The study was approved by the ethics committee of the University of Geneva. All participants gave informed consent prior to taking part in the study.

### 2.3 Materials

#### 2.3.1 Self‐reported procrastination: PPS

To assess self‐reported procrastination, participants completed the French version of the PPS (Rebetez et al., 2014), which consists of 11 items, evaluating two dimensions of procrastination: “voluntary delay” and “observed delay.” “Voluntary delay” describes actively putting off actions or decisions [e.g., “I delay making decision until it's too late”]. “Observed delay” comprises more unintentional or passive procrastination [e.g., “I don't get things done on time”]. Participants read each statement and indicated on a 5‐point Likert scale how accurately the statement describes them or their habits [1 = “very seldom or not true for me”; 5 = “very often true for me”].

#### 2.3.2 Procrastination behavior task

To assess procrastination behavior in a naturalistic situation, participants were assigned a specific task that had to be performed before a specific deadline. In detail, at the end of the final session of our study [see Section 2.4 for further information], students received a signed attendance sheet, confirming their participation in the study. They were then informed that in order to be allowed to participate in the final exam of the course and to subsequently validate the course credits [if they passed the exam], they had to scan and return the signed sheet via email before a specific date [i.e., before 12 a.m. of the day 1 week prior to the course exam]. In detail, participants were not instructed that they had to perform the task as fast as possible but that they had to send in the sheet before the specified deadline. This deadline was fixed so that all participants had at least 3 weeks to perform the task [for a different study setting a deadline of 3 weeks, see McCrea, Liberman, Trope, and Sherman (2008)]. This avoided that participants had to perform the task too urgently, and it allowed enough time to plan performing the task, but also to procrastinate. Due to practical restrictions [i.e., testing had to be spread over multiple weeks], the total number of days before the deadline could vary between 21 and 39 days between participants. The outcome measure was the number of days that elapsed before handing in the attendance sheeting. Note that if participants send a picture of their attendance sheet [instead of a proper scan], this also counted as valid response.

### 2.4 Procedure

All participants started the study with a laboratory session, during which they first gave informed consent, provided socio‐demographic information, filled out the PPS, and worked on a series of other cognitive tasks [as the present data stems from a larger study, also see Zuber, Ballhausen, Haas, Cauvin, Da Silva Coelho, Daviet, Ihle, and Kliegel (2020)]. Overall, the laboratory session lasted for approximately 30 min. At the end of the laboratory session, participants received a signed sheet confirming their participation, and they were instructed on the procrastination task [i.e., having to send a scan of the sheet before the end of the semester via email in order to validate their course credits].

## 3 RESULTS

### 3.1 Calculating self‐report scores for “voluntary delay” and “observed delay”

To calculate each participant's level of self‐reported “voluntary delay” and “observed delay,” we applied Bartlett's factor score approach (Bartlett, 1937; also see DiStefano, Zhu, & Mîndrilă, 2009) based on the two factors suggested by previous studies, namely Factor 1 = “voluntary delay” and Factor 2 = “observed delay” (see Rebetez et al., 2014). Bartlett's factor score approach calculates a standardized score for each participant, which situates the participant in relation to the group [group mean = 0; positive scores indicating that the participant scored above the mean, negative scores indicating scores below the mean]. Compared with other methods [such as sum‐scores or overall averaging], this approach only takes into account items that load on a common factor, resulting in more reliable estimates of actual factor scores (see DiStefano et al., 2009).^1^

### 3.2 Descriptive statistics and correlations of outcome measures

As “voluntary delay” and “observed delay” of the PPS are standardized factor scores, they both had an overall *M* = 0 and *SD* = 1. The correlation of the two factors was *r* = .46, *p* ≤ .001, indicating two distinguishable yet strongly related factors of procrastination. The mean procrastination behavior score [number of days for which the task was postponed] was *M* = 15.46 [*SD* = 11.12], whereas the median was *Mdn* = 13.50. Participants' procrastination behavior significantly correlated with both self‐reported factors: *r*_voluntary delay_ = .41, *p* ≤ .001; *r*_observed delay_ = .47, *p* ≤ .001.

### 3.3 Examining the relation between procrastination self‐reports and behavior

We conducted a multiple predictors regression analysis to examine whether the two self‐reported factors predicted participants' procrastination behavior. This revealed that self‐reports significantly explained 26.8% of variance in procrastination behavior [*F*(2,89) = 16.29, *p* < .001]. Specifically, procrastination was predicted by “voluntary delay” [*β* = .25, *t* = 2.49, *p* = .015] as well as by “observed delay” [*β* = .35, *t* = 3.43, *p* < .001], indicating that both self‐reported dimensions predicted how many days participants postponed a naturalistic task.

## 4 DISCUSSION

The present study is one of the first to examine whether self‐reported levels of procrastination relate to participants' actual procrastination behavior using a contextualized everyday life task as behavioral marker. To this end, we assessed procrastination via a self‐reported questionnaire that has been suggested to reliably capture procrastination [i.e., PPS], and we applied an objective measure of procrastination behavior by assigning participants a naturalistic task that they could typically encounter in real‐life [i.e., instructing students to send back an attendance sheet before a specific deadline]. Results show that self‐reported levels of procrastination explained a significantly large portion of variance in participants' actual behavior. In detail, both subdimensions of the PPS—that is, “voluntary” and “observed” delay were significant predictors of the number of days participants postponed performing the send‐back task.

These findings have both theoretical as well as clinical implications. In terms of measurement validity, the first goal of the current study was to examine whether procrastination self‐reports assessed via the PPS could accurately reflect participants' actual procrastination behavior. Validating a self‐assessed questionnaire can have several advantages: in clinical contexts, for example, it may be easier to ask individuals to fill in a brief questionnaire rather than instructing them to perform a real‐life task, which would imply verifying after how much time they performed that task, and so forth. Similarly, both for clinicians and for researchers, it is advantageous that questionnaire data are available immediately, whereas a behavioral task would require waiting for several weeks or months until the procrastination task would be [or would still not be] performed. In this regard, our findings suggest that self‐reports on the PPS represent a valid and reliable proxy of individuals' actual procrastination behavior, which should help researchers and clinicians to justify the use of the PPS rather than a more complex behavioral task.

In terms of processes and mechanisms that underlie procrastination, the second goal of the current study was to assess these two different yet related subdimensions of the PPS would differentially predict procrastination behavior. Our findings show that procrastination behavior was positively linked to both factors of self‐assessment, namely, to participants' tendency to voluntarily delay scheduled tasks as well as to a more passive notion of frequently running out of time or of being bad at meeting deadlines.

From a theoretical viewpoint, these findings indicate that when an opportunity to perform a planned task occurs, different mechanisms may lead to procrastination. In certain situations, people may voluntarily decide to delay task‐execution [e.g., “I'd rather do this later and do something else now”]. In view of previous literature, this can have different reasons. Sometimes, individuals may procrastinate in order to avoid the immediate negative consequences related to performing the task. Specifically, they may procrastinate because they want to avoid unpleasant feelings related to the task or because they are afraid of failing at the task and therefore—by avoiding to perform the task—aim to protect their self‐esteem (Steel, 2010). Other times, individuals may procrastinate because they see an advantage in the postponement and therefore use procrastination as conscious strategy to tackle everyday tasks. In this context, studies suggest that individuals voluntarily delay certain tasks because this increases pressure toward the deadline and thus boosts their motivation (Chu & Choi, 2005). Thereby, procrastinators aim to be more efficient, to gain time, to be less undecided and more productive (e.g., Choi & Moran, 2009; Hensley, 2014; Schraw, Wadkins, & Olafson, 2007).

Besides actively deciding to delay certain tasks, our findings further suggest that in other situations individuals may have a more passive perception of procrastination [e.g., “Although I really intended to do it before the deadline, I just couldn't manage to do it in time.”]. In this respect, our data showed that procrastination behavior was predicted by self‐reports of “observed delay”: participants who estimated that they generally are bad at meeting deadlines or that they frequently cannot find the time to do certain things waited longer before performing the behavioral task. Rather than perceiving themselves as the acting person that voluntarily decides to postpone a task, individuals with high scores on “observed delay” may feel as mere observers of how procrastination occurs and that—despite having the intention to perform a task—they seem unable to act on their intentions. Subjectively, they may have the impression of observing how time passes and how deadlines approach, without being able to do anything about it. In this context, it seems that procrastinators have difficulties with projecting themselves into their future and that they struggle to create rich, vivid mental images of upcoming events (Rebetez, Barsics, Rochat, D'Argembeau, & Van der Linden, 2016). As a consequence, they tend to be bad at premeditating and at anticipating potential consequences of their present actions (Sirois, 2004), which makes it difficult for procrastinators to manage and pursue their personal goals (e.g., Gustavson, Miyake, Hewitt, & Friedman, 2014).

Finally, our findings also have clinical implications in the context of understanding how procrastination behavior occurs and how one could aim to reduce procrastination. The fact that both subdimensions of procrastination self‐reports predicted participants' actual behavior supports that procrastination does not represent a simple, unidimensional construct but rather seems to be influenced by different factors (see Rebetez et al., 2014) [for a unifactorial model, see Steel (2010)]. Consequently, it is important to consider both facets that underlie its structure—particularly in the context of potential interventions. Our study suggests that on one side procrastination may be reduced by intervening on voluntary delay and associated factors. For example, interventions may aim at reducing impulsivity and at increasing self‐control. Similarly, targeting goal management and helping individuals to perform tasks as soon an opportunity to do so occurs [rather than choosing to delay]. For example, using implementation intentions may be useful, as they help individuals to better adhere to planned activities by determining a *when*, *where,* and *how* to perform a task [e.g., “when event X occurs, then I will do action Y"]. In this line, certain studies suggest that implementation intentions may help reduce procrastination (e.g., Owens, Bowman, & Dill, 2008; but also see Gustavson & Miyake, 2017). In the same line, training visual imagery could help individuals to create more vivid and concrete mental representations. This may increase premeditation and allow to be more realistic regarding the consequences of one's actions.

Conversely, our results suggest that procrastination may also be reduced by intervening on observed delay. For example, interventions might help individuals to more efficiently act on their intentions [instead of passively observing how deadlines pass] by targeting their planning skills, by training them to better organize their daily activities, or by improving their time management. Similarly, in view of the factors associated to observed delay, interventions to improve individuals' motivation, self‐esteem, and emotion regulation may prove beneficial. However, considering the novelty of our findings, their potential application remains hypothetical at present and future studies will have to investigate in detail if such interventions can effectively reduce procrastination.

To conclude, considering the limited literature available today, present findings indicate that the PPS forms a useful tool that can assess real‐life procrastination behavior more reliably than other questionnaires. The few previous studies on real‐life procrastination had administered other scales and found rather small [to medium] correlations between self‐reports and behavior [typically below .30] (see e.g., Krause & Freund, 2014). Using the PPS, however, resulted in correlations that were of medium [to large] size [*r* = .41 and .47 for the two subdimensions], indicating that the PPS captures real‐life procrastination more appropriately. In addition, most of the previous studies did either not use an objective measure of procrastination behavior or provided inconclusive results regarding how well behavior was assessed via self‐reports [see Svartdal et al., 2018 for an overview]. Thus, our study critically extends the existing literature in that it (a) provides new insights in the relation between objective measures of procrastination behavior and participants' self‐reports and (b) demonstrates that the PPS represents a concise and valid tool to assess procrastination, which better represents and predicts individuals' real‐life behavior.

### DECLARATION OF INTEREST STATEMENT

The authors declare no potential conflict of interest.

## Endnote

1 Note that an exploratory data analysis indicated that Item 4 did not allow to distinguish between the two dimensions of procrastination [as it had high loadings both on “voluntary delay” (= .44) as well as on “observed delay” (= .49)] and therefore was removed before calculating the final factor scores.
